# Systematic Control of Epoxidation in Low-cis Hydroxyl-Terminated Polybutadiene (HTPB) and Its Impact on Polyurethane Properties

**DOI:** 10.3390/polym18010039

**Published:** 2025-12-23

**Authors:** Sungyoung Yoon, Jongbok Lee

**Affiliations:** Department of Biological and Chemical Engineering, Hongik University, Sejong 30016, Republic of Korea; c4393202@g.hongik.ac.kr

**Keywords:** low-cis HTPB, epoxidized HTPB (EHTPB), polyurethane, adhesive, epoxidation

## Abstract

Hydroxyl-terminated polybutadiene (HTPB) is widely used in polyurethane binders, adhesives, and elastomers, but its low polarity and unsaturated backbone limit adhesion and long-term stability. Epoxidation presents a promising approach to addressing these limitations. However, most prior studies have focused on high-cis polybutadiene (PB), and systematic tuning of epoxidation in industrial low-cis HTPB has not been thoroughly examined. In this work, the epoxidation conversion of low-cis HTPB was systematically controlled by varying the equivalent amount of 3-chloroperbenzoic acid (*m*-CPBA). Conversion was governed solely by oxidant stoichiometry, while reaction time, concentration, and temperature had minimal effect, consistent with rapid, mixing-controlled epoxidation. Selective modification of 1,4-cis and 1,4-trans units enabled direct evaluation of how epoxidation degree influences polyurethane network formation and performance. Polyurethanes derived from epoxidized HTPB (EHTPB-PU) exhibited a clear correlation between epoxidation degree and network formation. Mechanical, adhesion, and chemical-resistance measurements revealed optimal performance at 10% epoxidation, where polarity and network compactness are effectively balanced. At this level, polyurethanes showed enhanced tensile strength, broad substrate adhesion, and increased resistance to acidic, basic, polar, and nonpolar environments, along with reduced water uptake. These results identify moderate epoxidation as a practical and efficient strategy for improving HTPB-based polyurethane materials.

## 1. Introduction

Hydroxyl-terminated polybutadiene (HTPB) is a representative liquid telechelic polymer bearing terminal –OH groups and is widely used in solid propellant binders, high-performance adhesives, potting compounds, elastomers, and coating materials due to its low glass transition temperature (T_g_ ≈ −80 °C), low surface energy, mechanical stability, abrasion resistance, and impact resistance [1,2,3,4,5,6,7]. The polymer backbone consists of 1,4-cis, 1,4-trans, and 1,2-vinyl units, and the relative ratio of these microstructures critically dictates viscosity, phase morphology, mechanical performance, and subsequent chemical reactivity. Typically, the cis configuration improves elasticity and elongation, whereas trans units reinforce strength and facilitate phase separation. In contrast, a higher vinyl content disrupts crystallinity, increases viscosity and T_g_, and suppresses microphase separation, resulting in reduced modulus and hardness [8,9,10].

**Scheme 1:**
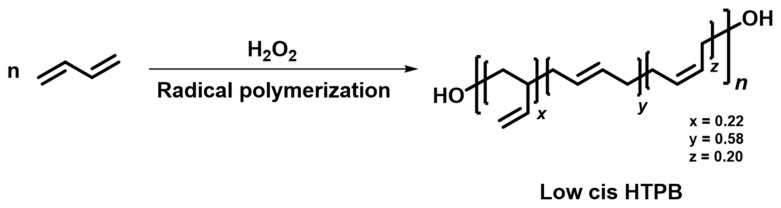
Typical synthetic method of industrial-grade low cis HTPB.

Industrial-grade HTPB is typically prepared via free-radical polymerization of 1,3-butadiene using hydrogen peroxide, a process that inherently yields a microstructure rich in 1,4-trans and 1,2-vinyl units and comparatively low in 1,4-cis content (Scheme 1) [11]. The microstructure, combined with the telechelic hydroxyl groups, strongly influences the subsequent polyurethane network structure. The terminal hydroxyl groups readily react with a wide variety of isocyanates to form polyurethane networks, enabling HTPB-based polyurethanes (HTPB-PUs) to exhibit excellent low-temperature flexibility, viscoelasticity, abrasion resistance, impact resistance, and thermal stability [12,13]. Despite these advantages, HTPB remains highly nonpolar, which limits its compatibility with polar media and energetic additives and restricts wetting and interfacial affinity toward polar or hydrophilic surfaces [14,15]. In addition, HTPB contains a high density of unsaturated double bonds that are intrinsically susceptible to oxidative and photochemical degradation, ultimately leading to deteriorated mechanical integrity, adhesion, and chemical resistance.

To overcome these limitations, several modification strategies have been explored [4,15,16,17,18]. A classical approach is the hydrogenation of HTPB, which saturates the double bonds to produce hydrogenated HTPB (HHTPB) [19,20,21]. Hydrogenation effectively suppresses UV-induced and oxidative degradation and significantly enhances long-term durability. Researchers have explored approaches that modify the unsaturated backbone of HTPB. However, because hydrogenation retains the nonpolar backbone, HHTPB still faces challenges in applications requiring strong interactions with polar components. In contrast, epoxidation of HTPB offers a dual benefit by reducing the degradation associated with unsaturated double bonds while simultaneously introducing polar functionality. Selective epoxidation converts the unsaturated double bonds into epoxy rings, affording epoxidized HTPB (EHTPB), which retains the flexibility and processability of the parent polymer with enhanced polarity. This increased polarity improves compatibility with polar additives, strengthens interfacial adhesion, and enhances chemical and moisture resistance. Moreover, the reduced unsaturation contributes to improved environmental stability and thermal durability. As a result, EHTPB provides a versatile platform for designing advanced polyurethane networks with enhanced mechanical strength, adhesion, and stability [14,15,22,23,24,25].

Various epoxidation methods have been explored for modifying polybutadienes (PB) and HTPB, and previous studies have predominantly relied on hydrogen peroxide-based oxidants in combination with phase-transfer catalysts. However, the conversion and selectivity in these systems are highly sensitive to the specific catalyst, the oxidant composition, and the rate of in situ peracid generation [26,27,28,29,30,31]. As a result, the epoxidation outcome can vary widely with reaction temperature, mixing efficiency, and reaction time, making reproducible and quantitative conversion control difficult. These limitations highlight the importance of developing a more selective and predictable epoxidation approach. Furthermore, most epoxidation strategies have been demonstrated primarily on high-cis PB and high-cis HTPB (1,4-cis contents > 95%) [31,32,33]. However, high-cis HTPB is typically prepared through multistep routes in which high-cis PB is first synthesized, then epoxidized, and subsequently cleaved to introduce hydroxyl terminal groups [31,34,35,36,37,38]. The cost and complexity of these procedures significantly restrict their feasibility for industrial use [39,40]. In contrast, low-cis HTPB, manufactured directly on a large scale through well-established radical polymerization processes, is the grade predominantly used in commercial polyurethane, binder, and adhesive formulations [1,37,41]. Nevertheless, detailed studies examining the epoxidation behavior of low-cis HTPB, including conversion control, microstructure-dependent reactivity, and the influence of epoxidation on the properties of the resulting polyurethane networks, remain limited. Establishing a systematic and controllable epoxidation strategy directly for industrial low-cis HTPB is therefore of substantial value for both research and industrial applications. Moreover, comprehensive analyses of how epoxidation conversion affects the structural, rheological, interfacial, and mechanical characteristics of low-cis HTPB-PUs are still insufficient, even though such insight is critical for rational material design.

In this study, we precisely controlled the epoxidation conversion of low-cis HTPB through systematic variation of 3-chloroperbenzoic acid (*m*-CPBA). In addition, the influence of epoxidation conversion on the structural and physical characteristics of EHTPB and the properties of the corresponding polyurethane networks was investigated to guide the rational design of high-performance materials.

## 2. Materials and Methods

HTPB (Mn = 2900 g/mol relative to PB standard, Evonik, Essen, Germany, 1,4-cis = 20%, 1,4-trans = 58%, 1,2-vinyl = 22%, Mean OH functionality = 2.4), *m*-CPBA (69–75%, Daejung, Siheung, Republic of Korea), sodium bicarbonate (Extra pure, Daejung, Siheung, Republic of Korea), chloroform (99.5%, SAMCHUN, Seoul, Republic of Korea), polymeric 4,4′-methylene diphenyl diisocyanate (PDI, NCO content = 30.0–32.0%, KUMHO MITSUI CHEMICALS CORP, Seoul, Republic of Korea), Tin(II) 2-ethylhexanoate (92.5–100%, Sigma-Aldrich, St. Louis, MO, USA), Carbon black (particle size = 22 nm, surface area = 134 m^2^/g, DBP absorption = 100, pH value = 3.5, Daemyung chemical, Hwaseong, Republic of Korea), CaCO_3_ (98%, Daejung, Siheung, Korea), 1,4-butanediol (99%, Sigma-Aldrich), polydimethylsiloxane (Sigma-Aldrich), CaO (Sigma-Aldrich). Plastic substrates were purchased from PLTIK, Incheon, Republic of Korea: Epoxy (EPO), acrylonitrile–butadiene–styrene (ABS), polycarbonate (PC), acrylic (ACR), polyethylene (PE), and polypropylene (PP).

### 2.1. Synthesis of EHTPB

HTPB (10 g, 3.44 mmol) was dissolved in chloroform (50 mL for ≤30% conversion, 100 mL for >30% conversion) and cooled in an ice bath for 10 min. *m*-CPBA (1.30–37.8 g, 7.50–219.25 mmol, corresponding to 2.5–78% epoxidation) was slowly added to the solution, and the ice bath was then removed. The reaction mixture was stirred at room temperature for 30 min. The mixture was cooled to 0 °C, and the *m*-CPBA precipitated upon cooling was removed by filtration. The resulting filtrate was washed with 10% aqueous NaHCO_3_ (3 times), the combined organic layer was dried over MgSO_4_, filtered through Celite, and concentrated under reduced pressure to afford the target-conversion EHTPB as a colorless viscous liquid.

### 2.2. Preparation of Neat Polyurethane Sheets

HTPB or EHTPB was vacuum dried under stirring to remove residual moisture. After drying, PDI (15 wt% relative to HTPB or EHTPB) was added, and the mixture was thoroughly mixed. The well-mixed prepolymer was poured into a PTFE mold and thermally cured at 80 °C for 19 h, followed by a post-cure at ambient temperature for 24 h to afford the polyurethane sheets of HTPB-PU or EHTPB-PU.

### 2.3. Preparation of Filled Polyurethane Sheets

HTPB or EHTPB was vacuum dried under stirring to remove residual moisture. After drying, a filler blend composed of carbon black (65 wt%), polydimethylsiloxane (1 wt%), CaCO_3_ (130 wt%), CaO (2 wt%), 1,4-butanediol (22 wt%), and tin(II) 2-ethylhexanoate (0.1 wt%) relative to the HTPB or EHTPB was added, and the mixture was thoroughly mixed at 120 °C for 50 min. The mixture was then allowed to cool to ambient temperature for approximately 10 min. Subsequently, PDI (15 wt% relative to HTPB or EHTPB) was added and mixed until a homogeneous prepolymer was obtained. The resulting mixture was poured into a PTFE mold and cured at 80 °C for 19 h, followed by a post-cure at ambient temperature for 24 h to afford the filled polyurethane sheets of HTPB-PU-F or EHTPB-PU-F.

### 2.4. Preparation of Shear Test Specimens

Shear test specimens were prepared according to ASTM D1002 [42]. HTPB or EHTPB was vacuum dried under stirring to minimize residual moisture. After drying, PDI (15 wt% relative to HTPB or EHTPB) was added, and the mixture was thoroughly mixed. The resulting prepolymer was applied to the bonding area (25 mm × 25 mm) between two plastic substrates, and the substrates were pressed together to prevent bubble formation. While maintaining pressure, the specimens were thermally cured at 80 °C for 19 h, followed by a post-cure at ambient temperature for 24 h.

### 2.5. Determination of Epoxidation Conversion

The epoxidation conversion was calculated using Equation (1) [43]. It was determined by comparing the proton intensity of the newly formed epoxy rings with the total proton intensity originally associated with the carbon–carbon double bonds (i.e., the sum of the residual double bond signals and epoxy ring signals after reaction). In this calculation, a correction factor of 0.5 was applied to the intensities of the vinyl epoxy and vinyl double bond peaks, since each trans and cis double bond corresponds to two protons, whereas the vinyl group contains three protons. Thus, the factor of 0.5 was introduced to normalize the 4.85–5.05 ppm vinyl signals to a two-proton equivalent.

To quantitatively evaluate the epoxidation conversion, the NMR integration windows were assigned as follows:

- 1,2-vinyl double bonds: 5.50–5.75 ppm (1H);
- 1,2-vinyl double bonds: 4.85–5.05 ppm (2H);
- 1,4-cis/trans double bonds: 5.25–5.50 ppm (2H);
- trans epoxy: 2.8–3.0 ppm (2H);
- cis epoxy: 2.55–2.75 ppm (2H);
- vinyl epoxy: 3 ppm (1H) and 2.5 ppm (2H).

(1) $$\frac{0.5\times I(2.5\;ppm)+I\left(2.55-2.75\;ppm\;\right)+I\left(2.8-3\;ppm\right)}{0.5\times I(2.5\;ppm)+I\left(2.55-2.75\;ppm\right)+I\left(2.8-3\;ppm\right)+0.5\times I\left(4.85-5.05\;ppm\right)+I\left(5.25-5.75\;ppm\right)}\times 100\;=Epoxidation\;conversion\;\left(\%\right)$$

### 2.6. Characterization

^1^H and ^13^C NMR spectra were obtained on a 400 MHz JEOL JNM-ECZ400S spectrometer (Tokyo, Japan)at room temperature. Chemical shifts are reported in ppm relative to the signals corresponding to the residual non-deuterated solvents (CDCl_3_: δ 7.26 for ^1^H and 77.16 for ^13^C at room temperature).

FT-IR spectra were acquired using a SHIMADZU IR Spirit-X spectrometer (Kyoto, Japan) equipped with a DTGS detector in ATR mode over the range of 400–4000 cm^−1^, with a resolution of 4 cm^−1^ and 45 scans per sample. Liquid samples were directly applied to the ATR crystal (diamond), and measurements were conducted under ambient conditions.

Mechanical properties were evaluated using a universal testing machine (UTM, VT-1T, Vluchem IND, Guri, Republic of Korea). Tensile testing was conducted according to ASTM D412 type-3 [44], using dumbbell specimens tested at a crosshead speed of 500 mm/min at room temperature. Shear strength was measured in accordance with ASTM D1002 [42] at a crosshead speed of 1 mm/min at room temperature.

Chemical resistance of polyurethane specimens was evaluated following ASTM D543 [45] with minor modifications. Specimens were cut into 1 cm × 1 cm pieces and immersed in separate vials containing 10% H_2_SO_4_ (acid), 30% NaOH (base), ethanol (polar solvent), and toluene (nonpolar solvent). After 7 days of immersion at ambient temperature, the samples exposed to 10% H_2_SO_4_ and 30% NaOH solutions were rinsed thoroughly with distilled water until no color change was observed upon contact with pH paper. The remaining surface moisture was carefully removed using a clean tissue, and the specimens were dried in an oven at 80 °C for 24 h. The samples immersed in ethanol and toluene were wiped to remove residual solvent and subsequently dried in an oven at 80 °C for 24 h. The dried samples were then weighed to determine the change in mass.

The water absorption behavior was evaluated following ASTM D570 [46] with minor modifications. Polyurethane specimens (1 cm × 1 cm) were immersed in deionized (D.I.) water. After immersion for 24 h at 80 °C, the samples were wiped to remove residual moisture and dried in an oven at 80 °C for 24 h. The dried samples were then weighed to determine the change in mass.

## 3. Results and Discussion

### 3.1. Synthesis of EHTPB

**Scheme 2:**
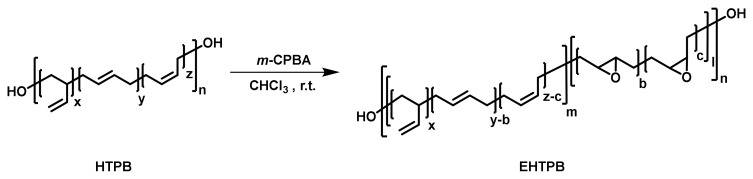
Synthesis of EHTPB [28,43].

To establish conditions for preparing a wide range of epoxidation conversions, the epoxidation behavior of HTPB was first examined under different reaction conditions, including molar concentration, reaction time, reaction temperature, and the molar equivalent of *m*-CPBA (Scheme 2).

**Figure 1:**
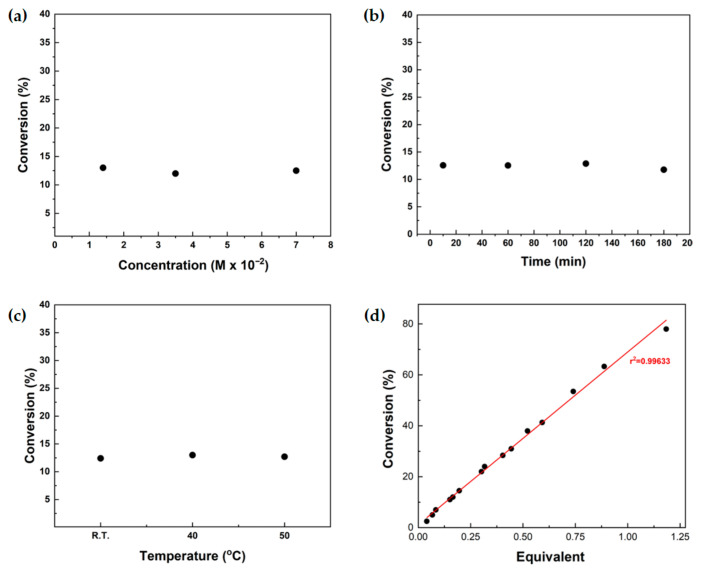
Epoxidation conversion of low-cis HTPB under various reaction conditions. Default reaction conditions: 0.07 M (≤30% conversion) or 0.035 M (>30% conversion), temperature = r.t., reaction time = 30 min, *m*-CPBA equivalents = 0.17 (12% conversion). Effect of (**a**) concentration, (**b**) reaction time, (**c**) reaction temperature, and (**d**) *m*-CPBA equivalent.

The effect of molar concentration was evaluated by carrying out the epoxidation of HTPB in chloroform at 1.4 × 10^−2^ M (1 g HTPB in 25 mL of CHCl_3_), 3.5 × 10^−2^ M (1 g HTPB in 10 mL of CHCl_3_), and 7.0 × 10^−2^ M (1 g HTPB in 5 mL of CHCl_3_) (Figure 1a). Although decreasing solvent volume generally increases reactant collision frequency and can enhance reaction efficiency, no significant difference in conversion was observed. These results indicate that the reaction does not proceed through concentration-dependent kinetics but is instead dominated by the instantaneous consumption of *m*-CPBA upon mixing. It is worth noting that at concentrations above 7.0 × 10^−2^ M, the addition of *m*-CPBA caused vigorous boiling and partial coagulation, preventing proper epoxidation. The influence of reaction time was assessed by conducting the reaction for 10 min, 1 h, 2 h, and 3 h (Figure 1b), and the conversion remained unchanged across all durations, indicating that the epoxidation is effectively completed during the initial mixing period. Reaction temperature was then examined at room temperature, 40 °C, and 50 °C (Figure 1c), and again, no notable differences in conversion were observed.

Finally, conversion was controlled by varying the molar equivalent of *m*-CPBA relative to HTPB (Figure 1d). Increasing the oxidant amount produced a nearly linear increase in conversion, reaching a maximum of 78%. Importantly, the maximum conversion corresponds closely to the combined fraction of cis and trans double bonds in the HTPB backbone, indicating that all accessible 1,4-double bonds were epoxidized. The remaining unsaturation originates from 1,2-vinyl units, which possess significantly higher activation energy (90.75 kJ mol^−1^) compared to 1,4-trans (31.35 kJ mol^−1^) and 1,4-cis (19.72 kJ mol^−1^) configurations, which makes them unreactive under the present conditions [28].

Such behavior is consistent with the well-established kinetics of *m*-CPBA epoxidation, which proceeds through a concerted Prilezhaev mechanism characterized by a highly reactive transition state and a very low activation barrier [47,48]. As a result, epoxidation occurs essentially upon contact between *m*-CPBA and the accessible double bonds, making the process diffusion- or mixing-controlled rather than kinetically controlled. Accordingly, reaction variables such as concentration, time, and temperature have minimal influence on conversion, and the extent of epoxidation is almost entirely determined by the amount of *m*-CPBA available for reaction.

In addition to the small-scale reactions, a large-scale epoxidation of HTPB was also performed to demonstrate the scalability of the reaction conditions (Table 1). Using 110 g of HTPB, the epoxidation conversion was reliably controlled by adjusting the *m*-CPBA equivalent (0.07, 0.21, and 0.43 equiv.), yielding 5%, 15%, and 30% EHTPB, respectively, consistent with small-scale results. These results confirm that the epoxidation process is stoichiometry-driven and readily scalable without loss of conversion accuracy.

### 3.2. Structural Analysis of EHTPB

**Figure 2:**
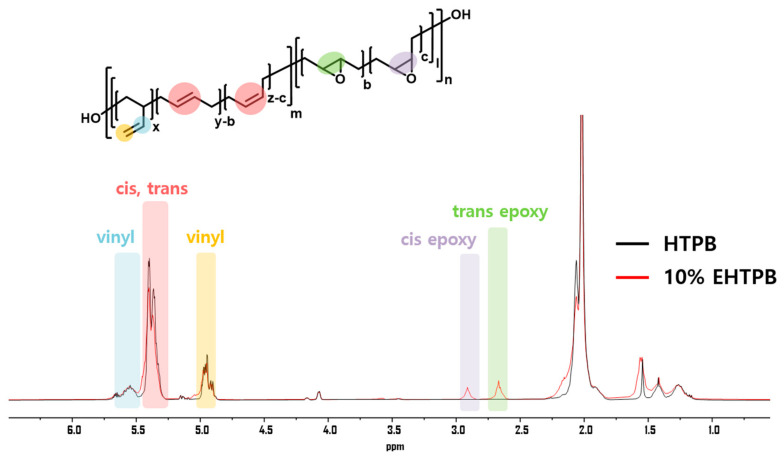
^1^H NMR spectra of HTPB (black) and 10% EHTPB (red) in CDCl_3_.

The characteristic chemical shifts of HTPB before and after epoxidation have been well established in previous studies, and the spectra obtained in this work were consistent with those reports [27,30,43]. As shown in Figure 2, epoxidation resulted in the appearance of new signals at 2.7–2.9 ppm and 2.55–2.75 ppm, corresponding to the trans-epoxy protons and cis-epoxy protons, respectively. These newly formed peaks provide direct evidence that the *m*-CPBA successfully converted the 1,4-cis and 1,4-trans double bonds into epoxy rings.

**Figure 3:**
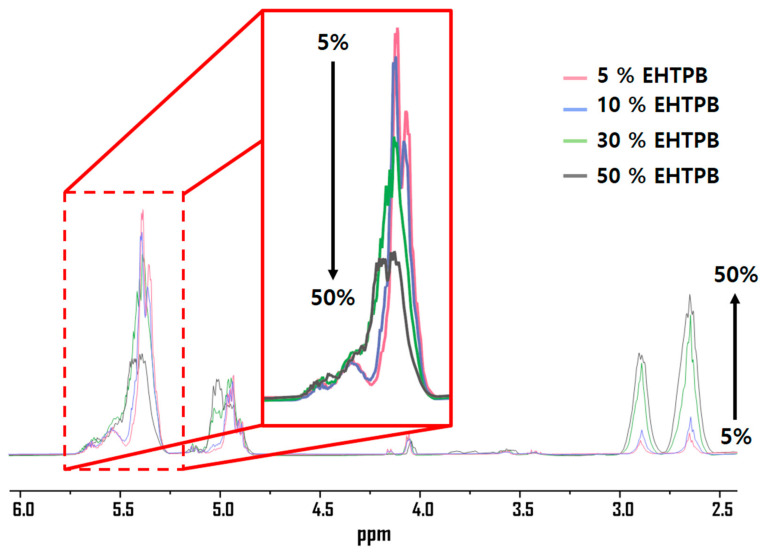
^1^H NMR spectra of EHTPB at different epoxidation conversion (5%, 10%, 30%, and 50%) in CDCl_3_, normalized to the 1,2-vinyl protons at −5.0 ppm. Signals at 2.8–3.0 ppm and 2.55–2.75 ppm correspond to trans- and cis-epoxy protons, respectively, while the resonance at 5.25–5.5 ppm is assigned to 1,4-cis/trans olefinic protons.

These results can be clearly observed by comparing the spectra of EHTPB with different epoxidation conversions (5%, 10%, 30%, and 50%) (Figure 3). As the conversion increased, the intensities of the cis/trans double-bond peaks decreased, while the corresponding epoxy-ring proton peaks increased, confirming that the amount of *m*-CPBA directly controlled the extent of epoxidation. Notably, the vinyl signals remained nearly unchanged, demonstrating that only 1,4-cis and 1,4-trans units underwent epoxidation under these reaction conditions, while 1,2-vinyl groups remained unreacted due to their significantly higher activation energy [28,43].

**Figure 4:**
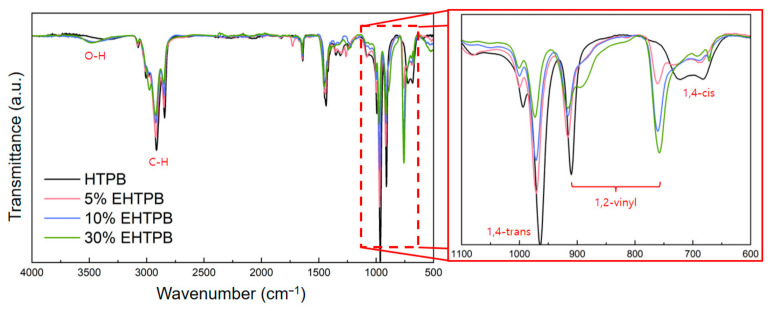
FT-IR spectra of HTPB and EHTPB at different epoxidation conversions (5%, 10%, and 30%). The full range spectra (**left**) and the magnified fingerprint region (1100–600 cm^−1^, **right**).

The FT-IR spectra of HTPB and EHTPB at varying epoxidation conversions (Figure 4) showed characteristic absorption bands corresponding to 1,4-trans (963 cm^−1^), 1,2-vinyl (910 cm^−1^), and 1,4-cis (680–730 cm^−1^) units [31,49]. Based on the ^1^H-NMR results, vinyl groups were expected to remain largely unreacted during epoxidation. Therefore, the 910 cm^−1^ peak should remain nearly constant, while the 1,4-cis, 1,4-trans peaks would be expected to decrease. In practice, as the conversion increased, the 1,4-trans and 1,4-cis units decreased, consistent with the expected consumption of these double bonds. However, the 1,2-vinyl bands at 910 cm^−1^ also appeared to decrease, which does not align with the ^1^H NMR results, indicating that the vinyl groups remain unreacted under the present conditions. Instead, a new absorption band at 750 cm^−1^ emerged and increased in intensity with increasing conversion. This behavior can be explained by changes in the local environment of the pendant vinyl groups. As epoxidation introduces rigid epoxy rings into the backbone, rotational freedom is reduced, and the chains become stiffer, causing the vinyl groups to adopt more restricted and preferentially oriented conformations [50]. Such conformational ordering enhances out-of-plane bending modes associated with the vinyl groups, resulting in increased intensity at 750 cm^−1^ and an apparent attenuation of the 910 cm^−1^ band due to the changes in local polarity and dipole environment rather than chemical consumption. Thus, the observed spectral features reflect reorganization of vinyl groups in a stiffer polymer matrix rather than their participation in the epoxidation reaction.

### 3.3. Analysis and Performance of EHTPB-PU

**Figure 5:**
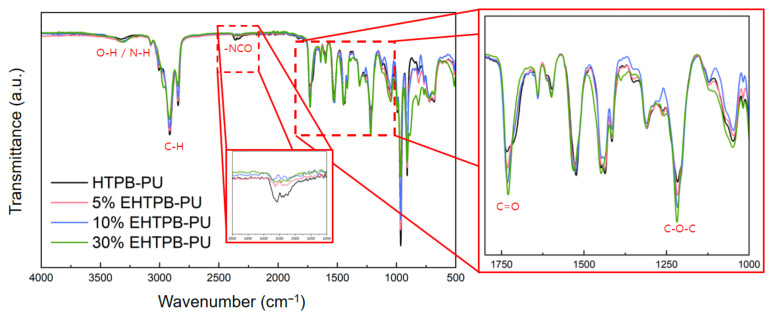
FT-IR spectra of HTPB-PU and EHTPB-PU at different epoxidation conversions (5%, 10%, and 30%). The full-range spectra (**left**) and the magnified region (1800–1000 cm^−1^, **right**).

**Figure 6:**
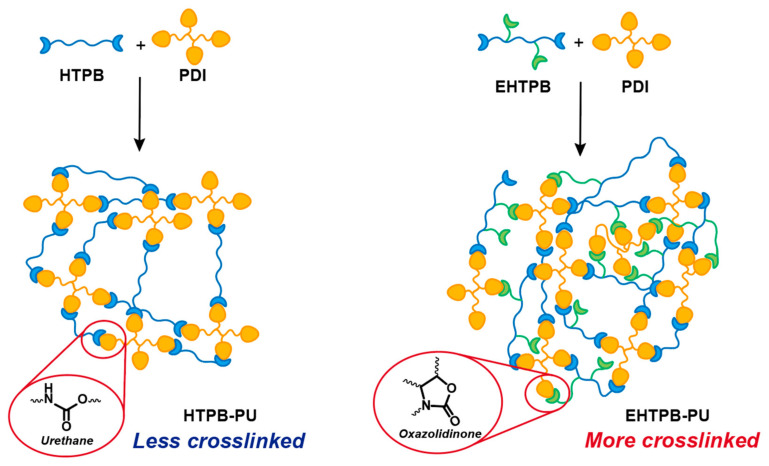
Schematic illustration of crosslinking differences between HTPB-PU and EHTPB-PU networks.

FT-IR analysis of polyurethane films prepared from HTPB and EHTPB with PDI (Figure 5) revealed clear structural evolution as epoxidation conversion increased. In neat HTPB-PU, the isocyanate absorption at 2350 cm^−1^ remained partially visible after curing, whereas in EHTPB-PU, this band nearly disappeared, despite identical NCO feed ratios. This additional consumption of NCO observed in EHTPB-PU resulted from secondary reactions between epoxides and isocyanates, affording oxazolidinone rings alongside conventional urethane linkages (Figure 6). As epoxidation conversion increased, strengthening of the C=O stretching (1730 cm^−1^) and C–O–C absorption (1217 cm^−1^) with increasing conversion further substantiates this behavior [15,51,52]. These observations collectively demonstrate that higher epoxide content increases the number of reactive sites available for network formation, leading to a greater crosslinking density within the polyurethane structure.

**Figure 7:**
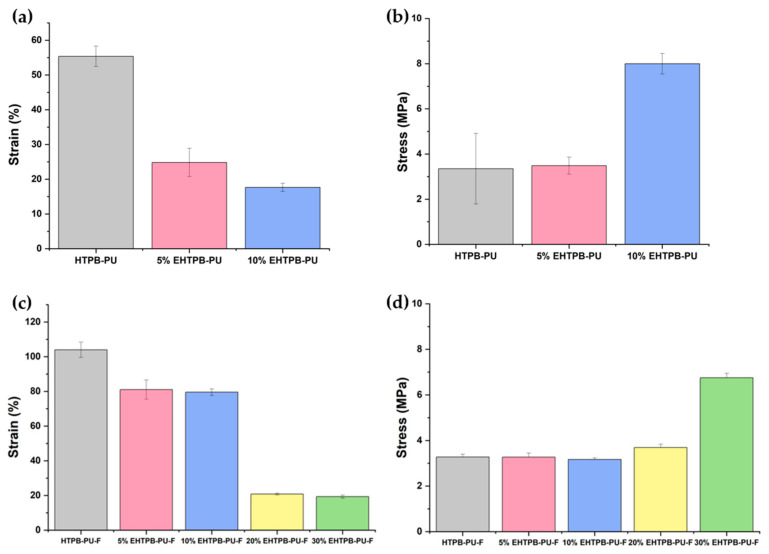
Mechanical properties of HTPB-PU and EHTPB-PU. Elongation at break: (**a**) HTPB-PU and EHTPB-PU; (**c**) HTPB-PU-F and EHTPB-PU-F. Tensile strength: (**b**) HTPB-PU and EHTPB-PU; (**d**) HTPB-PU-F and EHTPB-PU-F.

The consequences of these structural changes were also reflected in the mechanical behavior (Figure 7). Mechanical testing of neat polyurethanes prepared up to 10% epoxidation showed that even low conversions significantly altered material properties. The 10% EHTPB-PU, despite containing nearly twice the epoxide content, retained about 70% of the strain observed for 5% EHTPB-PU, reflecting a less steep reduction in strain at this conversion. In contrast, tensile strength increased much more substantially. HTPB-PU and 5% EHTPB-PU showed similar stress values, but the 10% EHTPB-PU exhibited an approximately 240% increase.

At 5% conversion, the elongation at break decreased to roughly 50% of unmodified HTPB-PU. In contrast, the 10% EHTPB-PU sample containing roughly twice the epoxy degree retained about 70% of the strain observed for 5% EHTPB-PU, indicating that the reduction in extensibility does not scale linearly with conversion. For tensile strength, HTPB-PU and 5% EHTPB-PU exhibited comparable stress values, whereas 10% EHTPB-PU showed a dramatic ~240% increase. These results suggest that the introduction of epoxy groups increases the crosslinking density due to epoxy–isocyanate reactions, leading to increased strength and reduced elongation. In addition, the epoxidation reaction progressively consumes the 1,4-cis double bonds that contribute to the inherent flexibility of the polybutadiene backbone. Their conversion into more rigid epoxy rings intrinsically decreases chain flexibility. It is worth noting that the viscosity of EHTPB increased substantially as the epoxidation conversion increased, trapping air during mixing and preventing efficient bubble release. These processing challenges became pronounced above 10% conversion, making it difficult to obtain homogeneous neat polyurethane films. Therefore, neat polyurethane samples were prepared only up to 10% EHTPB, while higher-conversion formulations were evaluated using a filled system (PU-F) to manage viscosity, improve mixture stability, and suppress bubble-related defects.

In the filled systems, 5% and 10% EHTPB-PU-F exhibited relatively high strain values (approximately 80%) compared to HTPB-PU-F, but a dramatic decrease in elongation to ~20% for the 20% and 30% EHTPB-PU-F. Meanwhile, tensile strength remained similar for HTPB-PU-F through 20% EHTPB-PU-F, but increased by nearly 200% at 30% EHTPB-PU-F. This difference from the neat polyurethane system can be attributed to the presence of a chain extender within the filler blend, which partially offsets the increase in crosslinking density at low conversion. As a result, the filled polyurethanes retain higher strain values up to 20% conversion, delaying the onset of network stiffening compared with the neat PU. In contrast, at higher conversions (≥20%), the increasing crosslinking density outweighs the chain-extending effect, leading to the observed increase in tensile strength and decrease in elongation. This progression indicates that excessive epoxidation ultimately produces an over-crosslinked, brittle network with limited deformability. Similar behavior has also been reported in epoxy resin and polyurethane systems, where epoxy content beyond an optimal range no longer improved mechanical strength and instead resulted in embrittlement [53,54,55].

**Figure 8:**
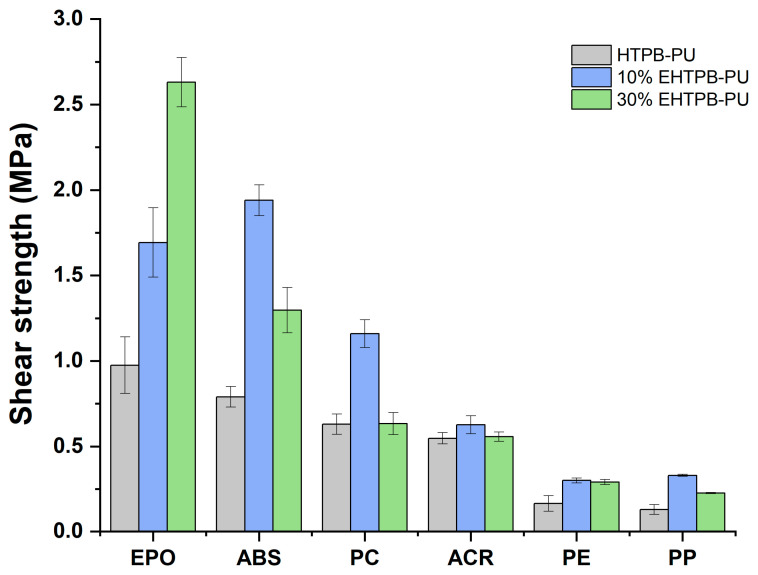
Shear strength of HTPB, 10% EHTPB, and 30% EHTPB polyurethane adhesives on various plastic substrates.

Adhesion performance also depended strongly on the degree of epoxidation, which is particularly important given that HTPB-PUs are widely used as potting, encapsulation, and coating materials where reliable bonding to diverse substrates is essential. Because introducing epoxide groups increases polymer polarity, an initial improvement in adhesion to polar plastics was expected. Adhesion to epoxy (EPO) substrates indeed increased most prominently, with the 30% EHTPB-PU displaying nearly 3 times higher shear strength than HTPB-PU (Figure 8). This substantial enhancement reflects the stronger polar interactions introduced by epoxidation and highlights the effectiveness of intrinsic backbone modification for bonding to highly polar surfaces. In contrast, for moderately polar substrates such as ABS, PC, and ACR, adhesion strength decreased at 30% conversion relative to 10% EHTPB. This behavior can be attributed to two factors: (1) at high conversions, the adhesive becomes more polar than the substrate, reducing interfacial compatibility, and (2) the high viscosity of 30% EHTPB likely produced microcracks or surface defects during application. Interestingly, improved adhesion was achieved even on nonpolar substrates, demonstrating the versatility of the epoxidation approach. For PE and PP, the enhancement is not due to polarity effects but to improved mechanical interlocking enabled by the moderately increased viscosity at 10% conversion.

Overall, these results indicate that for applications other than highly polar epoxy substrates, a moderate epoxidation level of approximately 10% provides the most favorable balance of viscosity, wetting, and cohesive strength, making it a broadly effective adhesive formulation across substrates with varying polarity. In contrast to such external, interface-driven approaches, such as surface treatments or nanofiller functionalization [56,57], the present results showed that tuning the intrinsic epoxidation conversion of the polymer backbone provided a direct and effective strategy for modulating adhesion [53,58].

### 3.4. Chemical and Moisture Resistance of EHTPB-PU

**Figure 9:**
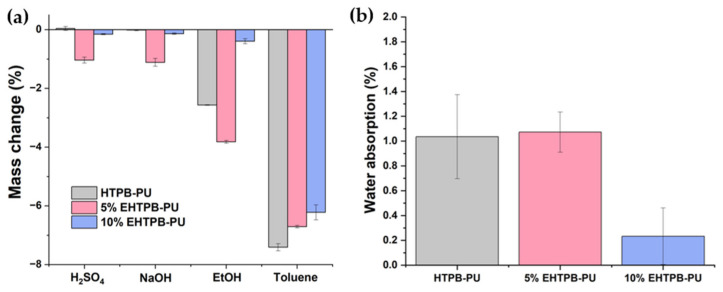
(**a**) Mass change of HTPB-PU, 5% EHTPB-PU, and 10% EHTPB-PU after immersion in 10% H_2_SO_4_, 30% NaOH, ethanol, and toluene for 7 days at ambient temperature. (**b**) Water absorption of HTPB-PU, 5% EHTPB-PU, and 10% EHTPB-PU after immersion in D.I. water at 80 °C for 24 h.

Chemical resistance tests were performed on HTPB-PU and EHTPB-PU under acidic (10% H_2_SO_4_), basic (30% NaOH), polar solvent (EtOH), and nonpolar solvent (toluene) environments (Figure 9a). Under acidic and basic conditions, EHTPB-PUs generally showed slightly higher mass loss than HTPB-PU. However, the degradation of the 10% EHTPB-PU sample was lower than that of the 5% EHTPB-PU sample and was nearly comparable to that of HTPB-PU. This trend suggests that small amounts of epoxide may initially increase susceptibility to chemical attack, but additional epoxide incorporation strengthens the network by increasing crosslinking density, thereby improving chemical stability.

In ethanol, a polar solvent capable of hydrogen bonding with urethane groups, an increase in mass change was initially expected with increasing epoxidation conversion due to the increased polarity of the polyurethane network. Consistent with this expectation, the 5% EHTPB-PU sample exhibited a slightly higher mass loss than unmodified HTPB-PU, which can be attributed to increased polymer–solvent interaction that facilitates ethanol penetration and extraction of residual low-molecular-weight species. However, further increasing the epoxidation conversion led to a markedly different behavior. The 10% EHTPB-PU sample showed a significantly reduced mass loss, indicating that the formation of a more compact and tightly crosslinked network at this conversion effectively suppresses solvent penetration and limits the extractable fraction. In contrast, when exposed to the nonpolar solvent toluene, the extent of mass change progressively decreased with increasing epoxidation conversion. This behavior can be attributed to the increased polarity of the polymer matrix, which reduces its affinity toward nonpolar media and enhances resistance to toluene.

Water absorption measurements (Figure 9b) also showed a similar trend. Although higher epoxidation levels increase polymer polarity and would therefore be expected to enhance water uptake, the 10% EHTPB polyurethane showed the lowest water absorption. These results demonstrate that chemical resistance toward acidic, basic, solvent, and aqueous environments is governed by a balance between polymer–solvent interactions and network structure, with moderate epoxidation providing the most effective resistance across a range of chemical and aqueous environments.

## 4. Conclusions

This study demonstrated that a systematic and scalable strategy for tailoring the structure and properties of industrial low-cis HTPB through controlled epoxidation. The epoxidation proceeded in a predictable and selective manner, targeting 1,4-cis and 1,4-trans units while leaving the 1,2-vinyl units unreacted. Importantly, the extent of conversion was governed almost entirely by oxidant stoichiometry, rather than reaction time, concentration, or temperature. Such behavior is consistent with rapid, mixing-controlled Prilezhaev epoxidation, enabling reliable and reproducible control of conversion, even at larger reaction scales. This precise control enabled direct investigation of how epoxidation degree governs the polyurethane network.

Systematic evaluation of mechanical, adhesion, and chemical-resistance properties revealed that the incorporation of epoxide groups substantially influenced crosslinking behavior of the resulting polyurethanes. Formation of oxazolidinone linkages and increased crosslinking density contributed to higher tensile strength but lower elongation. In terms of adhesion, an optimal region appeared near approximately 10% epoxidation, where polarity, viscosity, and interfacial contact were balanced most effectively across substrates of varying polarity, except for the highly polar epoxy substrate, for which adhesion continued to increase at higher conversions. Chemical resistance testing further demonstrated that the 10% EHTPB-PU exhibited the greatest stability toward acidic, basic, polar, and nonpolar environments. In addition, water-uptake measurements showed that the same conversion level resulted in the lowest absorption, reflecting a favorable combination of polarity and network compactness.

These findings highlight that controlled, moderate epoxidation provides a scalable and practical pathway to significantly enhance the performance and durability of HTPB-derived polyurethane materials.

## Figures and Tables

**Table 1:** Large-scale epoxidation of HTPB.

HTPB	*m*-CPBA	Equivalent *	Target Conversion (%)	Conversion (%)
110 g (37.9 mmol)	25.0 g (144.9 mmol)	0.07	5	5
110 g (37.9 mmol)	75.1 g (435.0 mmol)	0.21	15	15
110 g (37.9 mmol)	150.2 g (870.4 mmol)	0.43	30	30

* Molar equivalent of *m*-CPBA with respect to the C=C double bonds of HTPB.

## Data Availability

The original contributions presented in this study are included in the article. Further inquiries can be directed to the corresponding author.

## References

[1] Gopala Krishnan P.S., Ayyaswamy K., Nayak S.K. (2013). Hydroxy Terminated Polybutadiene: Chemical Modifications and Applications. J. Macromol. Sci. Chem. A.

[2] Quagliano Amado J.C., Ross P.G., Mattos Silva Murakami L., Narciso Dutra J.C. (2022). Properties of Hydroxyl-Terminal Polybutadiene (HTPB) and Its Use as a Liner and Binder for Composite Propellants: A Review of Recent Advances. Propellants Explos. Pyrotech..

[3] Tu J., Xu H., Liang L., Li P., Guo X. (2020). Preparation of high self-healing efficient crosslink HTPB adhesive for improving debonding of propellant interface. New J. Chem..

[4] Yoon S., Lee S., Lee J. (2024). Comprehensive Review on Post-Polymerization Modification of Hydroxyl-Terminated Polybutadiene (HTPB). Elast. Compos..

[5] Zhang W., Zhang G., Du L., Zhang C., Li L., Zhu J., Pei J., Wu J. (2018). Synthesis of hydroxyl-terminated polybutadiene bearing pendant carboxyl groups by combination of anionic polymerization and blue light photocatalytic thiol-ene reaction and its pH-triggered self-assemble behavior. React. Funct. Polym..

[6] Zhou W., Zuo J. (2013). Mechanical, thermal and electrical properties of epoxy modified with a reactive hydroxyl-terminated polystyrene-butadiene liquid rubber. J. Reinf. Plast. Compos..

[7] Rahman E.U., Khan A., Humayun M., Khan M., Shah N., Rehman N., Shah L.A., Khan M.S., Bououdina M. (2024). Preparation and characterization of hydroxyl-terminated polybutadiene graft ferrocene based composite. J. Polym. Res..

[8] Dey A., Sikder A.K., Athar J. (2017). Micro-structural effect on hydroxy terminated poly butadiene (HTPB) prepolymer and HTPB based composite propellant. Nanomed. J..

[9] Shankar R.M., Roy T.K., Jana T. (2009). Terminal functionalized hydroxyl-terminated polybutadiene: An energetic binder for propellant. J. Appl. Polym. Sci..

[10] Pournaghshband Isfahani A., Shahrooz M., Yamamoto T., Muchtar A., Ito M.M., Yamaguchi D., Takenaka M., Sivaniah E., Ghalei B. (2021). Influence of microstructural variations on morphology and separation properties of polybutadiene-based polyurethanes. RSC Adv..

[11] Prine N. (2018). Characterization and Selection of Hydroxyl-Terminated Polybutadiene Polymers for High-Strain Applications. Bachelor’s Thesis.

[12] Li J., Ning Z., Yang W., Yang B., Zeng Y. (2022). Hydroxyl-Terminated Polybutadiene-Based Polyurethane with Self-Healing and Reprocessing Capabilities. ACS Omega.

[13] Zhang X., Zheng J., Fang H., Zhang Y., Bai S., He G. (2018). High dimensional stability and low viscous response solid propellant binder based on graphene oxide nanosheets and dual cross-linked polyurethane. Compos. Sci. Technol..

[14] Zhang Q., Shu Y., Liu N., Lu X., Shu Y., Wang X., Mo H., Xu M. (2019). Hydroxyl terminated polybutadiene: Chemical modification and application of these modifiers in propellants and explosives. Cent. Eur. J. Energ. Mater..

[15] Zhang X., Liu Y., Chai T., Ma Z., Jia K. (2020). Curing Reaction Kinetics of the EHTPB-Based PBX Binder System and Its Mechanical Properties. Coatings.

[16] Kim S., Lee M., Lee W.B., Choi S.-H. (2021). Ionic-Group Dependence of Polyelectrolyte Coacervate Phase Behavior. Macromolecules.

[17] Ma L., Zhu X., Zhang W., Zhang H., Wang J., Qu J. (2021). Study on the preparation and performance comparison of side-chain hydroxyl-terminated polybutadiene derivatives with narrowly molecular weight distribution used for polyurethane. Polym. Test..

[18] Touidjine S., Boulkadid M.K., Trache D., Akbi H., Guettiche D., Belkhiri S., Nourine M. (2023). Review on energetic copolymer binders for propulsion applications: Synthesis and properties. J. Polym. Sci..

[19] Sekkar V., Krishnamurthy V.N., Jain S.R. (1997). Mechanical and Swelling Properties of Copolyurethanes based on Hydroxy-Terminated Polybutadiene and its hydrogenated analog. Propellants Explos. Pyrotech..

[20] Šabata S., Hetflejš J. (2002). Hydrogenation of low molar mass OH-telechelic polybutadienes catalyzed by homogeneous Ziegler nickel catalysts. J. Appl. Polym. Sci..

[21] He Y.N., Li Q., Zhu C., Li H., Zheng S., Xue Z., Hu Y. (2018). Synthesis and properties of thermoplastic polyethylene based polyurethanes (PE-PUs). J. Polym. Res..

[22] Hosseini S.R., Alavi Nikje M.M. (2023). Synthesis and characterization of novel epoxy-urethane coating and its graphene nanocomposites. Polym. Compos..

[23] Zhang Y., Wang G., Xu Y., Sun J., Zhang X., Zheng T., Zhang L. (2024). POSS/EHTPB synergistically toughened epoxy resin for cryogenic application. Polymer.

[24] Li H., Zhao L., Su K., Feng H., Wang D., Qu C. (2021). A comparative study on the rheological, thermal, and mechanical performance of epoxy resin modified with thermoplastics. J. Adhes. Sci..

[25] Zhang J., Zhang Z., Huang R., Tan L. (2025). Advances in Toughening Modification Methods for Epoxy Resins: A Comprehensive Review. Polymers.

[26] Nikje M.M.A., Mozaffari Z. (2007). Chemoselective epoxidation of hydroxyl-terminated polybutadiene (HTPB) using in-situ generated dimethyl dioxirane (DMD). Des. Monomers Polym..

[27] Wang Q., Zhang X., Wang L., Mi Z. (2009). Epoxidation of hydroxyl-terminated polybutadiene with hydrogen peroxide under phase-transfer catalysis. J. Mol. Catal. A Chem..

[28] Wang Q., Zhang X., Wang L., Mi Z. (2009). Kinetics of Epoxidation of Hydroxyl-Terminated Polybutadiene with Hydrogen Peroxide under Phase Transfer Catalysis. Ind. Eng. Chem. Res..

[29] Nikje M.M.A., Hajifatheali H. (2011). Nanoclay-Mediated Epoxidation of HTPB Using In-Situ-Generated Dimethyl Dioxirane. Des. Monomers Polym..

[30] Nikje M.M.A., Hajifatheali H. (2011). The role of cloisite 30B as a phase transfer catalyst (PTC) in polybutadiene epoxidation. Polym. Technol. Mater..

[31] Zhou Q., Jie S., Li B.-G. (2014). Preparation of Hydroxyl-Terminated Polybutadiene with High Cis-1,4 Content. Ind. Eng. Chem. Res..

[32] De Risi F.R., D’Ilario L., Martinelli A. (2004). Synthesis and characterization of epoxidized polybutadiene/polyaniline graft conducting copolymer. J. Polym. Sci. Part A Polym. Chem..

[33] Cao Z., Zhou Q., Jie S., Li B.-G. (2016). High cis-1,4 Hydroxyl-Terminated Polybutadiene-Based Polyurethanes with Extremely Low Glass Transition Temperature and Excellent Mechanical Properties. Ind. Eng. Chem. Res..

[34] Min X. (2019). A novel synthetic strategy for hydroxyl-terminated polybutadiene containing high cis-1,4 contents based on a Ni(ii)-coordinated anionic initiating system. RSC Adv..

[35] Zhu X., Fan X., Zhao N., Liu J., Min X., Wang Z. (2018). Comparative study of structures and properties of HTPBs synthesized via three different polymerization methods. Polym. Test..

[36] Meng D., Sang L., Zhang P., Deng J., Guo X. (2024). Properties of high cis-1,4 content hydroxyl-terminated polybutadiene and its application in composite solid propellants. Def. Technol..

[37] Min X., Fan X. (2019). A New Strategy for the Synthesis of Hydroxyl Terminated Polystyrene-b-Polybutadiene-b-Polystyrene Triblock Copolymer with High Cis-1, 4 Content. Polymers.

[38] Zhou Q., Jie S., Li B.-G. (2015). Facile synthesis of novel HTPBs and EHTPBs with high cis-1,4 content and extremely low glass transition temperature. Polymer.

[39] Kébir N., Campistron I., Laguerre A., Pilard J.-F., Bunel C., Couvercelle J.-P., Gondard C. (2005). Use of hydroxytelechelic cis-1,4-polyisoprene (HTPI) in the synthesis of polyurethanes (PUs). Part 1. Influence of molecular weight and chemical modification of HTPI on the mechanical and thermal properties of PUs. Polymer.

[40] Lee F., Radmall R., Heming J., Haddleton D., Lewtas K., McNally T. (2025). Hydroxyl-terminated polybutadienes (HTPB) and glycidyl azide polymer (GAP) as solid rocket propellant binders: A review of synthesis and properties. Eur. Polym. J..

[41] Vilar W.D., Moutinho M.T.M., Menezes S.M.C., Coutinho F.M.B. (1997). Characterization of hydroxyl-terminated polybutadiene. Polym. Bull..

[42] (2019). Standard Test Method for Apparent Shear Strength of Single-Lap-Joint Adhesively Bonded Metal Specimens.

[43] Aguiar M., de Menezes S.C., Akcelrud L. (1994). Configurational double bond selectivity in the epoxidation of hydroxy-terminated polybutadiene with m-chloroperbenzoic acid. Macromol. Chem. Phys..

[44] (2016). Standard Test Methods for Vulcanized Rubber and Thermoplastic Elastomers—Tension.

[45] (2021). Standard Practices for Evaluating the Resistance of Plastics to Chemical Reagents.

[46] (2018). Standard Test Method for Water Absorption of Plastics.

[47] Abdullin M.I., Basyrov A.A., Kukovinets O.S., Glazyrin A.B., Khamidullina G.I. (2013). Epoxidation of syndiotactic 1,2-polybutadiene with peracids. Polym. Sci. Ser. B.

[48] Kawahara E.M., Ogawa M., Yamato S., Yamaguchi T., Hori K., Sumimoto M. (2023). Theoretical and experimental mechanistic study of water molecule involvement in the Prilezhaev reaction. Phys. Chem. Chem. Phys..

[49] Wu K., Liu Y., Ma Y., Tan M., Chai T., Hu F., Liao L., Pan D., Liu C. (2021). Pretreatment of hydroxy-terminated polybutadiene (HTPB)/toluene diisocyanate (TDI) binder system for biodegradation. Adv. Compos. Hybrid Mater..

[50] Zuchowska D. (1980). Polybutadiene modified by epoxidation. 1. Effect of polybutadiene microstructure on the reactivity of double bonds. Polymer.

[51] Kordomenos P.I., Kresta J.E. (1981). Thermal stability of isocyanate-based polymers. 1. Kinetics of the thermal dissociation of urethane, oxazolidone, and isocyanurate groups. Macromolecules.

[52] Yeganeh H., Jamshidi S., Talemi P.H. (2006). Synthesis, characterization and properties of novel thermally stable poly(urethane-oxazolidone) elastomers. Eur. Polym. J..

[53] Jung K.-H., Min K.D., Lee C.-J., Park B.-G., Jeong H., Koo J.-M., Lee B., Jung S.-B. (2019). Effect of epoxy content in Ag nanoparticle paste on the bonding strength of MLCC packages. Appl. Surf. Sci..

[54] Yang J., He X., Wang H., Liu X., Lin P., Yang S., Fu S. (2020). High-toughness, environment-friendly solid epoxy resins: Preparation, mechanical performance, curing behavior, and thermal properties. J. Appl. Polym. Sci..

[55] Zhou W., Li X., Cao D., Li Y., Zhang C., Li Z., Chen F., Li T., Cai H., Dang Z.M. (2020). Simultaneously enhanced impact strength and dielectric properties of an epoxy resin modified with EHTPB liquid rubber. Polym. Eng. Sci..

[56] Kim S., Sung K.-S., Kim N. (2025). Surface Modification of Boron Nitride Particles and Its Effects on Thermal and Mechanical Properties of Epoxy/Benzoxazine Adhesives. Elast. Compos..

[57] Zhang Y., Hasegawa K., Kamo S., Takagi K., Takahara A. (2023). Surface modification for enhanced lap shear strength of the epoxy-bonded joints consisting of metallic adherents and similar/dissimilar materials. ACS Appl. Polym. Mater..

[58] Chailee O., Parnklang T., Mora P., Pothisiri T., Jubsilp C., Rimdusit S. (2020). Epoxy-based composite adhesives with improved lap shear strengths at high temperatures for steel-steel bonded joints. J. Appl. Polym. Sci..

